# A model for initiating research data management services at academic libraries

**DOI:** 10.5195/jmla.2019.545

**Published:** 2019-07-01

**Authors:** Kevin B. Read, Jessica Koos, Rebekah S. Miller, Cathryn F. Miller, Gesina A. Phillips, Laurel Scheinfeld, Alisa Surkis

**Affiliations:** NYU Health Sciences Library, New York University School of Medicine, New York, NY, kevin.read@nyumc.org; Health Sciences Library, Stony Brook University, jessica.koos@stonybrook.edu; Falk Library, Health Sciences Library System, University of Pittsburgh, Pittsburgh, PA, rebekah.miller@pitt.edu; Gumberg Library, Duquesne University, Pittsburgh, PA, millerc12@duq.edu; Hillman Library, University of Pittsburgh, Pittsburgh, PA, gap64@pitt.edu; Health Sciences Library, Stony Brook University, Stony Brook, NY, laurel.scheinfeld@stonybrook.edu; Health Sciences Library, New York University (NYU) School of Medicine, New York, NY, alisa.surkis@med.nyu.edu

## Abstract

**Background:**

Librarians developed a pilot program to provide training, resources, strategies, and support for medical libraries seeking to establish research data management (RDM) services. Participants were required to complete eight educational modules to provide the necessary background in RDM. Each participating institution was then required to use two of the following three elements: (1) a template and strategies for data interviews, (2) the Teaching Toolkit to teach an introductory RDM class, or (3) strategies for hosting a data class series.

**Case Presentation:**

Six libraries participated in the pilot, with between two and eight librarians participating from each institution. Librarians from each institution completed the online training modules. Each institution conducted between six and fifteen data interviews, which helped build connections with researchers, and taught between one and five introductory RDM classes. All classes received very positive evaluations from attendees. Two libraries conducted a data series, with one bringing in instructors from outside the library.

**Conclusion:**

The pilot program proved successful in helping participating librarians learn about and engage with their research communities, jump-start their teaching of RDM, and develop institutional partnerships around RDM services. The practical, hands-on approach of this pilot proved to be successful in helping libraries with different environments establish RDM services. The success of this pilot provides a proven path forward for libraries that are developing data services at their own institutions.

## BACKGROUND

The National Library of Medicine (NLM) has quickly become the epicenter for data science at the National Institutes of Health (NIH), and NLM Director Patricia Flatley Brennan has spoken of the importance of increasing librarians’ capacity to support the management of biomedical data [1]. There have been considerable efforts in recent years to address the need for research data management (RDM) training for librarians. These resources include online curricula, such as those developed by librarians from the University of North Carolina at Chapel Hill [2] and Harvard University [3], and an online synchronous RDM course for librarians offered by the National Network of Libraries of Medicine (NNLM) National Training Office (NTO) [4]. Librarians have also created RDM curricula specifically designed to be used by other librarians to train researchers, such as the New England Collaborative Data Management Curriculum [3, 5].

The development of training programs has addressed one aspect of preparing librarians to provide RDM services, but evidence suggests that training alone is not sufficient to enable librarians to move into this realm. One challenge for librarians is overcoming institutional barriers. Wittenberg, Sackmann, and Jaffe emphasized the importance of understanding an institution’s culture around RDM services [6]. For example, Purdue University Libraries’ evaluations of their professional development workshops on data curation for librarians found that despite librarians’ increased confidence in their knowledge and ability to connect with researchers, institutional barriers impeded putting this new knowledge into action [7]. Another challenge is making inroads with the research community. A survey administered by Read and Surkis through health sciences librarian email discussion lists in 2015 found that 67% of respondents (48/72) indicated that a barrier to teaching RDM was their lack of comfort in engaging researchers on the topic [8]. This finding is consistent with other reports from the literature about the difficulty of engaging researchers to make use of specialized library services [9–14].

The goal of this pilot program was to provide a cohort of librarians from six institutions with RDM training as well as strategies, resources, and support designed to address the challenges described above to help them initiate or improve data services at their institutions. The RDM training, strategies, and resources used for this program were based on work led by Surkis and Read at New York University Health Sciences Library (NYUHSL) and consisted of online RDM training modules [15] and the Teaching Toolkit to teach RDM [16], a protocol for conducting data interviews [17], and guidance for hosting a data series [18], as well as support and guidance from Surkis and Read.

## CASE PRESENTATION

### Pilot program overview

The aim of the program was to initiate or expand data services in libraries serving health sciences populations. To meet this aim, librarians from participating libraries were asked to gain foundational knowledge of RDM by taking the NYUHSL online RDM training modules and to achieve two of the three following goals: (1) establish lines of communication with their research communities using the NYUHSL data interview template, (2) teach an introductory RDM class with the Teaching Toolkit, or (3) conduct a focused marketing effort for a series of data classes. Communication channels and regular meetings were established to provide ongoing support for the cohort. The ideal outcome was that each institution would achieve all three goals, but because there was a nine-month timeline for the pilot, it was not realistic to expect that every participating library would be able to move forward quickly enough to achieve all three.

### Pilot participant recruitment

The pilot program was designed with the unit of participation being a library rather than an individual librarian. The motivation for this approach was a recognition of the difficulty that an individual librarian would have implementing data services if they did not have support from their colleagues and library leadership. To this end, the pilot program required that each participating library provide a letter of support for their participants’ time commitment to the pilot.

Six libraries responded to a call from the NNLM Middle Atlantic Region for pilot participants. Suitability for participation was determined by conducting a pre-pilot interview. These interviews were conducted by phone and consisted of a series of open-ended questions that assessed what the current state of their RDM services was, what their library hoped to gain from participating in the pilot, and what their level of institutional commitment was. Supplemental Appendix A provides pre- and post-pilot program interview questions. One interview was conducted for each institution, with between one and five librarians participating. These interviews indicated that there was a range in the level of RDM services offered across institutions (Table 1), with most interviewed librarians having a minimal to moderate knowledge of RDM prior to the pilot. Institutions identified several motivations for participation, including:

- enhancing the visibility of the library in the realm of data
- gaining a better understanding of how research is done in the institution
- developing partnerships and offering an additional service to ensure the production of high-quality research in their institution
- hitting the ground running with a suite of services that would enable them to conduct outreach and engage with their research communities
- learning from other librarians and colleagues with expertise and experience in this area
- expanding their knowledge related to institutional needs around data management

All six libraries were deemed suitable participants. The participants were University at Buffalo, University of Delaware, Drexel University, Duquesne University, Stony Brook University, and Temple University. Between two and eight librarians participated from each institution, with twenty-six librarians participating in total. Three of the institutions were standalone health sciences libraries, and three were university libraries serving biomedical research communities (Table 1). At each institution, librarians took on different roles, with some participating in all aspects of the pilot and others serving only an administrative oversight role.

**Table 1:**
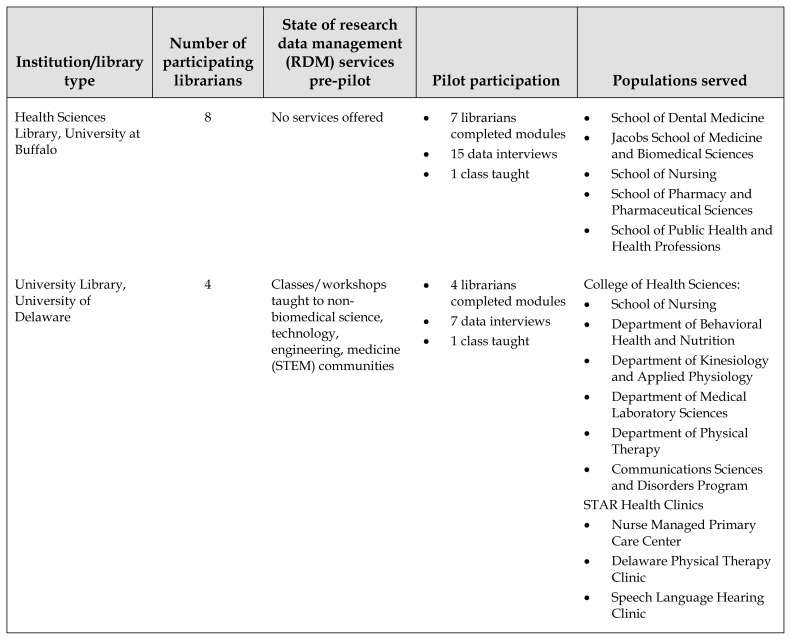

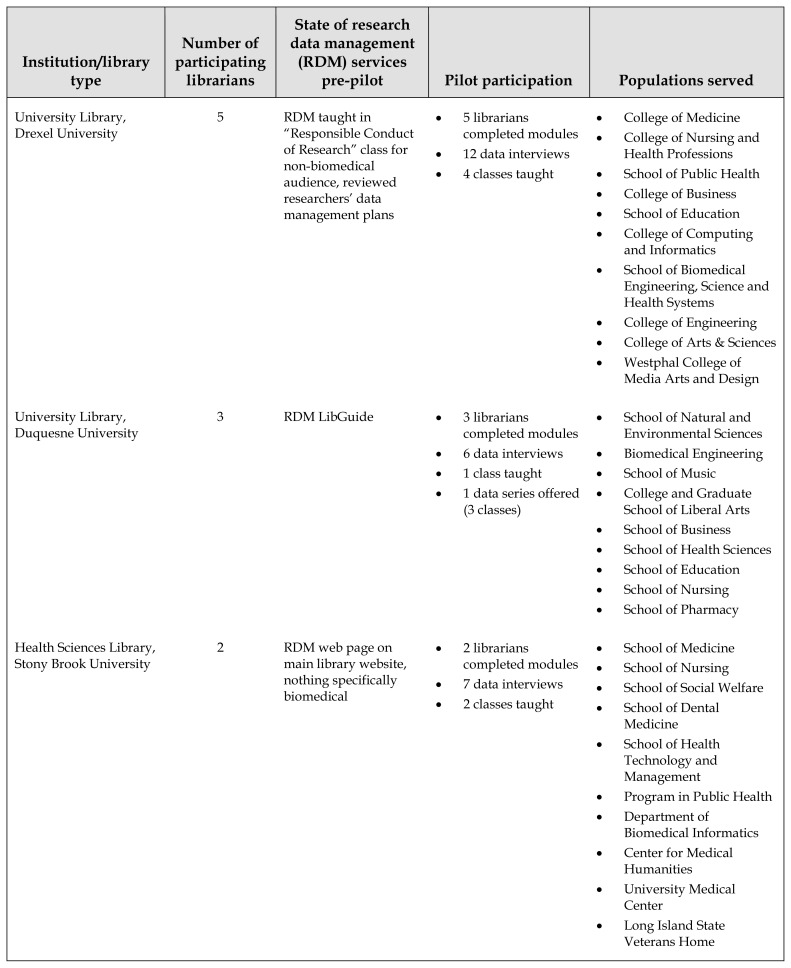
Overview of pilot institutions

Institution/library type	Number of participating librarians	State of research data management (RDM) services pre-pilot	Pilot participation	Populations served
Health Sciences Library, University at Buffalo	8	No services offered	7 librarians completed modules 15 data interviews 1 class taught	School of Dental Medicine Jacobs School of Medicine and Biomedical Sciences School of Nursing School of Pharmacy and Pharmaceutical Sciences School of Public Health and Health Professions
University Library, University of Delaware	4	Classes/workshops taught to non-biomedical science, technology, engineering, medicine (STEM) communities	4 librarians completed modules 7 data interviews 1 class taught	College of Health Sciences: School of Nursing Department of Behavioral Health and Nutrition Department of Kinesiology and Applied Physiology Department of Medical Laboratory Sciences Department of Physical Therapy Communications Sciences and Disorders ProgramSTAR Health Clinics Nurse Managed Primary Care Center Delaware Physical Therapy Clinic Speech Language Hearing Clinic
University Library, Drexel University	5	RDM taught in “Responsible Conduct of Research” class for non-biomedical audience, reviewed researchers’ data management plans	5 librarians completed modules 12 data interviews 4 classes taught	College of Medicine College of Nursing and Health Professions School of Public Health College of Business School of Education College of Computing and Informatics School of Biomedical Engineering, Science and Health Systems College of Engineering College of Arts & Sciences Westphal College of Media Arts and Design
University Library, Duquesne University	3	RDM LibGuide	3 librarians completed modules 6 data interviews 1 class taught 1 data series offered (3 classes)	School of Natural and Environmental Sciences Biomedical Engineering School of Music College and Graduate School of Liberal Arts School of Business School of Health Sciences School of Education School of Nursing School of Pharmacy
Health Sciences Library, Stony Brook University	2	RDM web page on main library website, nothing specifically biomedical	2 librarians completed modules 7 data interviews 2 classes taught	School of Medicine School of Nursing School of Social Welfare School of Dental Medicine School of Health Technology and Management Program in Public Health Department of Biomedical Informatics Center for Medical Humanities University Medical Center Long Island State Veterans Home
Health Sciences Library, Temple University	4	Taught “Research Data Management Essentials” using New York University Health Sciences Library (NYUHSL) Teaching Toolkit as part of NYUHSL National Institutes of Health (NIH) Big Data to Knowledge (BD2K) pilot	4 librarians completed modules 12 data interviews 5 classes taught 1 data series offered (5 classes)	School of Medicine School of Dentistry School of Pharmacy Temple University Hospital System College of Public Health

### Pilot program components and evaluation

#### Online modules

Librarians from each institution were required to take eight online modules [19] to develop a foundation of understanding for RDM. The online modules consisted of text, videos, and quizzes that addressed: (1) background information to provide a concrete understanding about different types of data and research processes; (2) introduction to the research data lifecycle; (3) differences between bench and clinical research processes, environment, and data management issues; (4) incentives and requirements for research data management; (5) best practices for workflow and variable documentation and file-naming conventions to facilitate effective data management; (6) introduction to discipline-specific data standards; and (7) methods for researchers to store, archive, and preserve their data. All participants from the pilot institutions who personally conducted data interviews or taught an RDM class completed all the modules.

#### Data interviews

Institutions used a set of data interview questions and an approach developed at NYUHSL [17]. The purpose of these interviews was to establish connections with the research community and gain a better understanding of researchers’ language, attitudes, and practices related to RDM. To that end, the questions were open-ended and designed to encourage a conversational tone. This was in contrast to other data interview templates, such as those from Purdue [20], that were designed to systematically investigate all facets of a researcher’s RDM practices.

Institutions used several methods for identifying researchers to interview, including (1) searching for researchers with active grants using National Institutes of Health (NIH) RePORTER and the National Science Foundation (NSF) funding lists, (2) mining college and department websites to identify junior and senior faculty, (3) collaborating with liaison librarians to target researchers with an existing relationship with the library, and (4) using lists of researchers who had participated in past surveys. Institutions that had the greatest success recruiting interviewees relied mainly on outreach to researchers who had previous relationships with the library. Challenges described included scheduling issues, the lack of preexisting relationships with researchers, and, for one institution, a requirement for institutional review board approval. Librarians from each institution recorded the number of interviews that they conducted and, optionally, the interviewee’s academic department. Each institution interviewed between six and fifteen researchers (Table 1).

#### The Teaching Toolkit

The Teaching Toolkit [16] provided institutions with slides, a script designed for a sixty- to ninety-minute introductory RDM class, and an evaluation form for class attendees. Institutions marketed the class in a variety of ways, including using their library’s class event web pages; sending emails to departments, faculty, and staff; writing promotional articles for their institutions; collaborating with their offices of science and research; using library multimedia (i.e., library screens with rotating announcements); and creating posters. One institution taught the class as part of a required “Responsible Conduct of Research (RCR)” course.

Institutions were encouraged to customize the Teaching Toolkit to better match their own teaching styles and meet their communities’ needs. Each institution made modifications, including adding institution-specific material and content (e.g., local information technology services, institutional repositories), condensing information about publisher and NIH mandates, creating self-evaluation checklists to help researchers identify their RDM status, adding NSF RDM requirements, and providing audience-specific examples. Institutions also introduced several hands-on activities such as case studies, worksheets, and exercises that asked researchers to draw their research workflows to increase interactivity.

**Figure 1:**
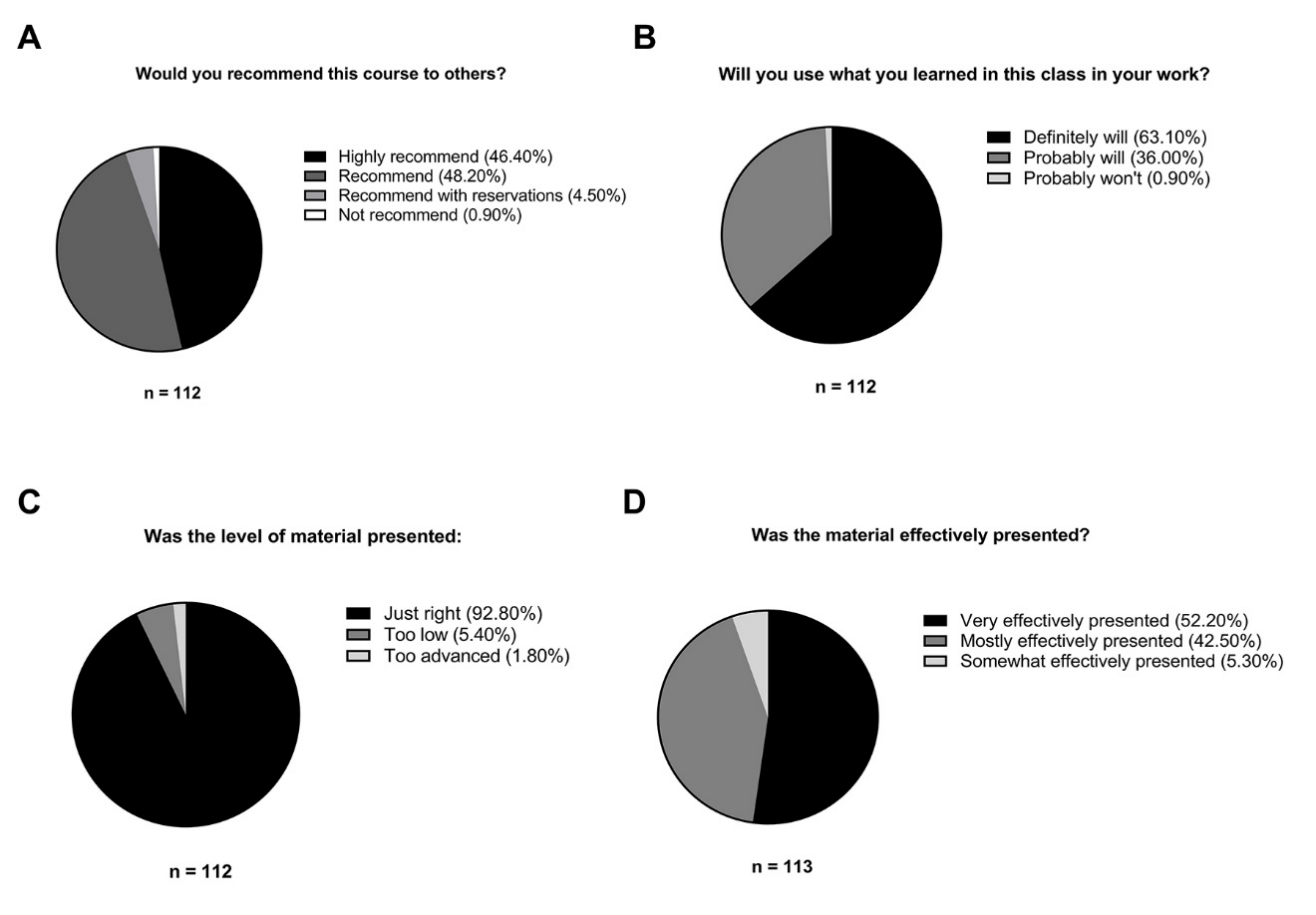
(A) Percentage of attendees’ course recommendations to others; (B) percentage of attendees’ indications of using what they learned in their work; (C) percentage of attendees’ ratings of the difficulty of presented material; (D) percentage of attendees’ ratings of the effectiveness of the instructors’ presentations

Librarians recorded the number of classes taught and number of attendees in each class. In addition, librarians from each institution were required to use the Teaching Toolkit evaluation form to collect data from the researchers who attended the class. Between 1 and 5 classes were taught to researchers at each institution, with a mean of 21 attendees per class (range, 4 to 50) and a total of 294 attendees across 14 classes. Supplemental Appendix B provides the data set with completed pilot program components for each participating institution. The total number of evaluation forms collected was 113; for classes taught as part of an RCR series, it was not always possible to disseminate class evaluations. Attendee evaluations at each institution were overwhelmingly positive (Figure 1). Supplemental Appendix C provides the data set of researcher reviews of the Teaching Toolkit class. In addition, 73% of attendees indicated an interest in advanced topics, including electronic lab notebooks, preservation, data ownership, data cleaning, data analysis, data privacy, data collection, and software tools.

#### Data series

The data series consisted of a branded series of classes taught over a short time period with instructors from within and outside of the library [17]. The series encouraged the librarians to collaborate with institutional partners and make use of a focused marketing effort. Institutions that hosted a data series recorded the names of classes taught, department of the instructor, number of attendees, and percentage of attendees who indicated that they would use what they learned in their current work. Two institutions organized a data series that was held during Love Data Week [21]. One institution collaborated with their Clinical Research Institute and Center for Bioethics, Urban Health, and Policy to provide REDCap and geographic information systems (GIS) classes, while the librarians taught an RDM class using the Teaching Toolkit, a data visualization course, and an additional hands-on RDM workshop [22]. At the second institution, the series consisted of the RDM class using the Teaching Toolkit, “Getting Started with SPSS,” and “Accessing Census Data,” all taught by librarians.

#### Pilot program support

Surkis and Read established communication channels through biweekly online meetings with all institutions and a Slack channel. Biweekly meetings included a round robin with pilot participants discussing their progress toward and approaches to implementing the different components of the pilot program, including outreach and marketing strategies and customizations of the Teaching Toolkit. Meetings also included presentations from NYUHSL librarians on additional data services offerings, such as REDCap, data visualization, and clinical research data management.

### Post-pilot program interviews

Post-pilot interviews (supplemental Appendix A) consisted of open-ended questions relating to the overall pilot program and its individual components. Interviews were conducted once for each institution, with between two and six librarians participating. These interviews took place within three weeks of the conclusion of the pilot and concentrated on identifying the perceived benefits of the entire pilot program as well as its individual elements, suggestions for improving the pilot program, and their institutions’ next steps for implementing RDM services. Typically, multiple librarians responded to each question, and responses were later summarized by Surkis and Read. There were no significant disagreements across the responses from librarians from a given institution.

Five of the six institutions indicated that the modules were very helpful, and one felt that they were only somewhat useful due to a lack of connection between the content of the modules and the questions asked in the data interviews. The biggest issue identified was that there were errors in the quizzes embedded in the modules, which have since been corrected.

All six institutions indicated that conducting data interviews was a valuable experience. Specifically, institutions indicated that the data interviews provided a well-defined purpose for setting a meeting with a researcher (n=5), formed new or strengthened existing relationships (n=4), and supplied information that they could use to inform future RDM services (n=4). While a formal analysis of the interview data was not a part of the pilot program, three institutions that undertook such an analysis indicated that it would have been helpful to have more support than was provided.

All six institutions indicated that the Teaching Toolkit was very helpful, pointing to the time saved over developing the content de novo, its ease of use, and its flexibility as particularly helpful. While many institutions felt that some aspects of the Teaching Toolkit did not fit their needs well (e.g., length, emphasis on certain topics, lack of interactive elements), all customized the content to remedy these perceived shortcomings.

The two institutions that hosted a data series indicated that it was helpful for marketing library RDM services. The institution that invited instructors from outside the library also indicated that the series allowed them to expand their course offerings, make or deepen connections with others at their institution, and initiate further teaching opportunities for the library.

Four institutions noted that the biweekly meetings helped keep them moving forward in implementing the pilot components, but every institution indicated that they felt there were shortcomings in the structure of the meetings. In particular, all indicated that the round robins were often not a productive use of time during the meetings and that Slack would have been a better forum for institutional updates. Four institutions noted the usefulness of NYUHSL librarians presenting other data curricula (e.g., data visualization, REDCap, clinical research data management) at the biweekly meetings. Another issue that five institutions identified was that the structure of communication was too top-down, with Surkis and Read communicating with each institution rather than the institutions communicating with each other.

All institutions indicated that they would continue offering versions of the Teaching Toolkit class and conduct outreach to new communities. Outreach strategies that were mentioned included organizing an RDM symposium, partnering with the Office of Research, and working with larger university steering committees. Four institutions indicated that they would like to further review the information gathered from their data interviews to identify service gaps and target potential partners. One institution has begun offering RDM consultations, while another has developed new data curricula with plans for developing more data courses and was in the process of hiring a research and data services librarian. Three institutions had plans to explore institutional partnerships to offer REDCap training. However, several barriers to continued service development were identified: four institutions mentioned lack of sufficient administrative or institutional support, two discussed insufficient knowledge of their institutional RDM landscape, and two felt the need for more in-depth training.

## DISCUSSION

This pilot program took a practical, hands-on approach to achieving the aim of offering a series of steps designed to enhance professional development, researcher relationships, and class offerings to support RDM in participating libraries. While the online RDM training modules contain only three to four hours of content, participants felt sufficiently comfortable with the material after taking the modules to engage with researchers by conducting data interviews and teaching RDM classes. This was particularly valuable for practicing librarians with little time for professional development. The data interviews, despite some issues that librarians had with data analysis, resulted in developing new connections with researchers and a better understanding of their RDM needs, skills, and interests. The Teaching Toolkit, with appropriate modifications, received positive evaluations across institutions, despite the fact that librarians were teaching material that they had not created themselves. Finally, the data series proved to be a useful marketing tool and an effective means of building and strengthening collaborations outside the library.

The pilot program achieved its aims: librarians at each institution took the modules, conducted data interviews, and taught the Teaching Toolkit class to their communities. Two institutions hosted a data series. Furthermore, the pilot program components had the intended results at each institution, and, in particular, the classes were uniformly positively reviewed. This is notable given the diverse environments of the institutions. The path forward for each institution is different and, in some cases, still to be determined. However, each institution is now offering at least a minimum level of RDM services, using the Teaching Toolkit for researcher RDM training and building on connections established with their respective research communities. Based on the participating institutions’ success with each program component, the authors believe the program materials—all freely available—are sufficient to achieve similar outcomes at other institutions. However, some may struggle to implement the program components in a timely manner without the community-based structure of the pilot.

The strength of the pilot program’s approach is that it provides both sufficient education and the critical push to begin engaging with the research community as opposed to strictly educational approaches [2, 3, 23]. A limitation of this program’s approach was that it relied upon librarians from one institution (NYUHSL) to guide the development of other libraries seeking to initiate RDM services. This approach raises two potential issues: (1) it provides a limited viewpoint as to how best to develop RDM services and (2) the time commitment required of the mentoring institution to support an active RDM community is not sustainable.

While the components of the pilot program are freely available, four of the institutions identified the structure and support that was offered in the pilot program as a key component of their impetus to move forward. Communities exist around health sciences data librarianship; there is a community of practice for data librarians (i.e., the Medical Library Association’s Data Special Interest Group) and a community for learners (e.g., NNLM NTO RDM class cohorts). There is not, however, a community explicitly designed to provide support and structure for librarians who are in the process of bringing the data skills that they have acquired to their work. The authors believe that more effective community building efforts would strengthen the approach described in this case report.

## SUPPLEMENTAL FILES

Appendix A Pre-pilot and post-pilot interview questions

Appendix B Data set with completed pilot program components for each participating institution

Appendix C Data set of researcher reviews of the Teaching Toolkit class
