# Correction

**DOI:** 10.5195/jmla.2020.947

**Published:** 2020-04-01

**VOLUME 107**

107(3) July, pages 439–40

Read KB, Koos J, Miller RS, Miller CF, Phillips GA, Scheinfeld L, Surkis A. A model for initiating research data management services at academic libraries. J Med Libr Assoc. 2019 Jul;107(3):432–41. DOI: http://dx.doi.org/10.5195/jmla.2019.545.

Pages 439–40: Acknowledgments of pilot program participants Deborah Chiarella, Pamela M. Rose, Aletia Morgan, and Gretchen Sneff are missing. The “Acknowledgments” should be:

This program was supported by the National Library of Medicine (NLM), National Institutes of Health (NIH), under cooperative agreement number UG4LM012342 with the University of Pittsburgh, Health Sciences Library System, and NIH R25LM012283. The content is solely the responsibility of the authors and does not necessarily represent the official views of the NIH. We acknowledge pilot participants of this program, specifically those from University at Buffalo: Nell Aronoff, Donna R. Berryman, AHIP, Deborah Chiarella, Amy Gische Lyons, AHIP, FMLA, Pamela M. Rose, Michelle L. Zafron, Elizabeth Stellrecht, and Linda Lohr; University of Delaware: Sarah E. Katz, Tom Melvin, Natalia Lopez, Sandra Millard, and Aletia Morgan; Drexel University: Abby L. Adamczyk, AHIP, Elizabeth Ten Have, Kathleen Turner, Janice Masud-Paul, and Deborah Morley; and Temple University: Jenny Pierce, Natalie Tagge, Gretchen Sneff, and Nancy Turner. The authors also thank Richard McGowan for discussion and comment on the manuscript.

