# Supplementary material for: A model for initiating research data management services at academic libraries
Source: J Med Libr Assoc. 2019 Jul 1;107(3):432–41. doi: 10.5195/jmla.2019.545 (PMC6579580; doi:10.5195/jmla.2019.545)
Supplement: Appendix A [file jmla-107-432-s001.docx]

# A model for initiating research data management services at academic libraries

## Kevin B. Read; Jessica Koos, AHIP; Rebekah S. Miller; Cathryn F. Miller; Gesina A. Phillips; Laurel Scheinfeld; Alisa Surkis

### APPENDIX A

### Pre-pilot and post-pilot interview questions

**Pre-pilot interview questions**

Can you tell us about the landscape of your institution?

- What schools do you serve?
- What existing partnerships do you have?
- Who are your strongest users?

What background do you (or your team have) in terms of training or work in data that you have done at your institution or elsewhere?

What has your institution done around data services?

- What worked?
- What didn’t work?
- What barriers have you encountered?
- If none, why? Anticipated barriers?

What would you like to gain out of the pilot program?

How many people/full-time equivalents (FTE) at your institution will be participating in the pilot?

What is your timeline for getting things started?

**Post-pilot interview questions**

What do you see in your institution’s future for offering data services—anything you want to expand on beyond what you put in the paper?

What value did you get out of the following and/or what changes would you suggest:

- Online modules
- Data interviews
- Teaching tool kit
- Data series planning
- Webinar/online community discussions

Looking back, what kinds of other support or guidance would have further facilitated the development of a data services program?

What would help you move your goals forward for implementing a data services program?
